
JOURNAL INFORMATION
==============================
NLM Title Abbreviation: Breast Cancer Res
Journal ID: Breast Cancer Res
NLM Journal ID: 100927353

Breast Cancer Research : BCR

ISSN: 1465-5411
EISSN: 1465-542X
Publisher: BMC

ARTICLE INFORMATION
==============================
PMCID: PMC5688493
DOI: 10.1186/s13058-017-0903-9
Article ID: 903
Article version: 1
Subjects: Meeting Abstracts

British Society of Breast Radiology Annual Scientific Meeting 2017

Dublin, Ireland. 5-7 November 2017

Electronic publication date: 2017 Nov 1
Print publication date: 2017
Volume: 19
Issue: Suppl 1
Issue sponsor: Publication of this supplement was funded by the British Society of Breast Radiology.
Electronic Location ID: 116
Copyright: © The Author(s). 2017
License: Open AccessThis article is distributed under the terms of the Creative Commons Attribution 4.0 International License (http://creativecommons.org/licenses/by/4.0/), which permits unrestricted use, distribution, and reproduction in any medium, provided you give appropriate credit to the original author(s) and the source, provide a link to the Creative Commons license, and indicate if changes were made. The Creative Commons Public Domain Dedication waiver (http://creativecommons.org/publicdomain/zero/1.0/) applies to the data made available in this article, unless otherwise stated.
License URL: https://creativecommons.org/licenses/by/4.0/

Conference name: British Society of Breast Radiology annual scientific meeting 2017
Conference Acronym: BSBR 2017
Conference location: Dublin, Ireland
Conference date: 5-7 November 2017
issue-copyright-statement: © The Author(s) 2017

==============================
Publisher’s Note

Springer Nature remains neutral with regard to jurisdictional claims in published maps and institutional affiliations.

